# Isolation and Characterization of Bovine RVA from Northeast China, 2017–2020

**DOI:** 10.3390/life11121389

**Published:** 2021-12-11

**Authors:** Xi Cheng, Wei Wu, Fei Teng, Yue Yan, Guiwei Li, Li Wang, Xiaona Wang, Ruichong Wang, Han Zhou, Yanping Jiang, Wen Cui, Lijie Tang, Yijing Li, Xinyuan Qiao

**Affiliations:** 1 Heilongjiang Key Laboratory for Animal Disease Control and Pharmaceutical Development, Department of Preventive, Veterinary Medicine, College of Veterinary Medicine, Northeast Agricultural University, Harbin 150038, China; 18008349515@163.com (X.C.); ww13936206166@163.com (W.W.); teng1085579571@163.com (F.T.); 13845115515@163.com (Y.Y.); wanglicau@163.com (L.W.); xiaonawang0319@163.com (X.W.); Zhouhan9659@163.com (H.Z.); jiangyanping2017@126.com (Y.J.); cuiwen_200@163.com (W.C.); tanglijie@neau.edu.cn (L.T.); yijingli@163.com (Y.L.); 2 Branch of Animal Husbandry and Veterinary of Heilongjiang Academy of Agricultural Sciences, Qiqihar 161000, China; hljslgyxh@163.com; 3 Department for Radiological Protection, Heilongjiang Province Center for Disease Control and Prevention, Harbin 150030, China; mice4@126.com

**Keywords:** bovine rotavirus, epidemiological characteristics, virus isolation, sequence analysis

## Abstract

Group A rotaviruses (RVAs) are major enteric pathogens causing infections in calves. To investigate the epidemiological characteristics and genetic diversity of bovine rotavirus (BRV), 233 fecal samples were collected from calves with diarrhea in northeast China. The samples were analyzed for sequences encoding the inner capsid protein VP6 (subgroup) and the outer capsid proteins VP7 and VP4 (G and P type, respectively) using RT-PCR. Ten of the 233 samples (4.3%) were identified as BRV positive and were used for virus isolation and sequence analysis, revealing that all strains analyzed were of the G6P[1] genotype. The isolates exhibited high VP6 sequence identity to the USA cow RVA NCDV strain (>99% amino acid identity) and were further shown to be closely related to Japanese cow RVA BRV101 and Israelian human RVA G6P[1] strains, with >99% amino acid identity to VP7 and VP4 proteins, respectively. Comparative analyses of genome-predicted amino acid sequences between the isolates and the NCDV strains indicated that the antigenicity and infectivity of the strains isolated had changed. In this study, BRV genotypes and the genetic diversity among vaccinated cattle herds were monitored to provide epidemiological data and references for early diagnosis, allowing for early detection of new, potentially pathogenic RVA strains.

## 1. Introduction

Rotaviruses (RVs), which are members of the *Reoviridae* family, are major causative pathogens of diarrhea in humans and animals, resulting in millions of childhood infections in developing countries [1,2]. The genomes of these viruses consist of 11 double-stranded (ds) RNA segments encoding six structural (VP1–4, VP6, and VP7) and five to six non-structural proteins (NSP1–5/6) [3]. Rotaviruses are classifified into nine groups (RVA–RVD; RVF–RVJ) (International Committee on Taxonomy of Viruses (ICTV). Available online: https://talk.ictvonline.org/taxonomy/ (accessed on 20 September 2021) based on the nucleotide sequence of the VP6 gene.

The RV virion is a triple-layered icosahedral particle. The outer capsid is composed of VP7 and VP4 proteins, which independently elicit neutralizing antibody responses. A dual classification system was established by differentiating between VP7 (G) and VP4 (P) genotypes [4], resulting in at least 41 G (glycoprotein antigen) and 57 P (protease-sensitive antigen) types according to the Rotavirus Classification Working Group (RCWG, 2017; https://rega.kuleuven.be/cev/viralmetagenomics/virus-classification (accessed on 6 December 2021).

BRVs are important causes of neonatal calf diarrhea (NCD), which is associated with high morbidity and mortality in dairy and beef cattle herds worldwide [5,6,7]. RVAs the main etiological factor identified especially in calves up to 30 days of age [8,9,10,11]. Vaccination of cows at the end of pregnancy is one of the main health management strategies for the control and prophylaxis of BRVA infections [7,12,13,14]. However, several studies have reported that the immune pressure promoted by mass vaccination can cause the emergence of new viral strains with distinct genotypes, and viral evolution can drive the occurrence of antigenic variants [15,16,17,18]. Some of these strains may be responsible for vaccine failure. For example, the occurrence of two RVA genotypes (G6P[11] and G10P[11]) in six G6P[5]-vaccinated dairy cattle herds has been reported in the southeast region of Brazil [19]. Furthermore, G6P[5] genotypes present in a BRVA field strain were identified as the causative agent of a diarrhea outbreak in a vaccinated beef cattle herd [11]. These genotypes were also distinct from the G and P types of BRVA strains covered by the commercial vaccine used to immunize the herd (G6P[1] and G10P[11]). Therefore, understanding RV diversity expansion and genetic exchange between strains will prove helpful for disease control [9,15,20,21]. 

Genetic analysis of RV sequences is thought to be the gold standard for investigating how the virus strains have evolved. Based on nucleotide (nt) and encoded amino acid sequences as well as with the help of phylogenetic analyses, genetic heterogeneity among G and P types of wild-type RVA strains can be assessed. Numerous RV genotypes have been isolated from calves, with at least ten different G types (G1,G2, G3, G5, G6, G8, G10, G11, G15, and G18) and six different P types (P[1], P[5], P[11], P[14], P[17], and P[21]) [22,23,24,25,26]. G6, G8, and G10 are the G types predominant in bovine isolates, whereas P[1], P[5], and P[11] have been recorded in the majority of cases of bovine diarrhea [16,25,26]. Despite China’s substantial dairy and beef cattle industries, there is limited information on the prevalence of BRV in Chinese cattle herds. Reports on the distribution of RV genotypes in China are rare [1,27]. In recent years, studies on BRV strains causing epidemic outbreaks in China have been carried out in Qinghai Province and the Inner Mongolia Autonomous Region; however, data on the prevalence of BRV remain scarce.

In this study, we collected the feces of calves from farms located in Heilongjiang and Jilin Provinces as well as in the Inner Mongolia Autonomous Region. All of the cattle herds included were regularly vaccinated against RV. The RV strains isolated from fecal samples were identified and characterized. The main structural genes were sequenced and employed for phylogenetic tree construction. The results obtained will help provide a better understanding of the evolutionary relationships between different RV strains, thus driving the development of vaccines and effective protection measures.

## 2. Materials and Methods

### 2.1. Sample Collection

**Figure 1:**
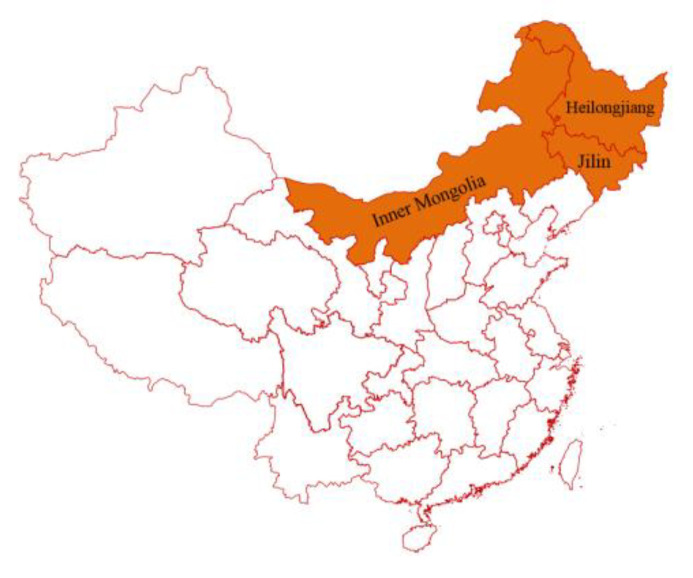
The map of the sampling regions.

A total of 233 bovine diarrhea samples were collected from cattle farms in Heilongjiang and Jilin Provinces and the Inner Mongolia Autonomous Region (Figure 1, Table 1). These samples were stored at −80 °C until further use for RNA extraction and virus isolation. The study was conducted between 2017 and 2020. 

### 2.2. Primers

The primers employed for detection of a *VP6* gene segment (211 bp in length) of BRV were designed using Primer 5.0 software based on the NCDV sequence of the BRV reference strain, which are referred to as BRV-1/BRV-2. Primers for amplification of full-length BRV *VP4* (2362 bp in length), *VP6* (1356 bp in length), and *VP7* (1062 bp in length) genes were designed using Primer 5.0 software based on the NCDV sequence of the BRV reference strain, which are referred to as BRV-VP4-F, BRV-VP4-R, BRV-VP6-F, BRV-VP6-R, BRV-VP7-F, and BRV-VP7-R (Table 2).

### 2.3. RT-PCR

Fecal samples were diluted 1:9 (*w*/*v*) in sterile phosphate-buffered saline (PBS) and centrifuged at 16,000× *g* (Thermofisher Heraeus Multifuge X1R) for 10 min. Viral RNAs were extracted from diluted fecal samples using the RNA Fast 200 total RNA extraction kit (Feijie Biotek, Inc., Shanghai, China) according to the manufacturer’s instructions. RNA samples were quantified using NanoVue Plus (Thermo Fisher Scientific, Waltham, MA, USA), and the purity of the RNA was assessed using the A260/280 ratio. The cDNA was synthesized from total RNA by using high-capacity cDNA reverse transcription kits (Applied Biosystems, Waltham, MA, USA). *VP6* gene fragments were amplified by RT-PCR using BRV-1/BRV-2 primers first to identify positive samples. The other common pathogens associated with NCD, including bovine parvovirus (BPV), bovine viral diarrhea virus (BVDV), and bovine coronavirus (BCoV), were also detected by PCR (results not shown). *VP4*, *VP6,* and *VP7* genes were then amplified by RT-PCR using primers specific for BRV-VP4-F, BRV-VP4-R, BRV-VP6-F, BRV-VP6-R, BRV-VP7-F, and BRV-VP7-R for subsequent sequencing analysis. The RT-PCR products were analyzed by electrophoresis on 1.5% agarose gels stained with ethidium bromide in TAE buffer (Beijing Solarbio Science & Technology Co., Ltd., Beijing, China). After electrophoresis at a constant voltage (100 V) for 30 min, the gel was visualized under UV light. PCR products were then purified, cloned into the pMD-19T simple vector (Takara Bio, Beijing, China), and sequenced. 

### 2.4. Cell Culture and Virus Isolation

Monkey kidney epithelial cells (MA104) were cultured in Dulbecco’s modified Eagle medium (DMEM) containing 10% (*v*/*v*) fetal calf serum (Gibco) at 37 °C in a humidified atmosphere with 5% CO_2_. The samples were filtered through a 0.22-μm filter (Millipore) and treated with trypsin at a final concentration of 25 μg/mL for 1.5 h at 37 °C. MA104 cells were incubated with the viral isolates for 1 h. The virus-containing supernatant was subsequently discarded and the cells were washed three times with PBS. Serum-free DMEM with a final trypsin concentration of 4 μg/mL was added, and cell cytopathic effects (CPEs) were examined. When approximately 80% of the cells displayed CPEs, whole-cell lysates were centrifuged and the supernatant was stored at −80 °C.

### 2.5. Immunoelectron Microscopy

Cell cultures infected with the ten strains isolated were collected and centrifuged to remove cell debris. PEG 8000 was then added to the supernatant to concentrate the virus. After centrifugation at 11,000× *g* for 30 min, the viruses were collected and resuspended in deionized water. Treated samples were mixed with an equal volume of anti-BRV serum (stored at our lab) and incubated overnight at 4 °C. Afterwards, the samples were centrifuged at 16,000× *g* for 1 h. The supernatants were discarded, and the precipitates were resuspended in 50 μL of deionized water for routine negative staining. The viruses were observed under a transmission electron microscope (Hitachi, Japan).

### 2.6. Indirect Immunofluorescence Assay

MA104 cells were inoculated with the isolated strains on a 24-well plate. After 36 h, the culture medium was discarded and the cells were washed three times with sterile PBS. Precooled absolute ethanol was added for fixation, and the cells were then washed three times with PBS. Subsequently, the cells were incubated with 0.2% Triton X-100 for 15 min to enable permeabilization. The cells were subsequently washed three times with PBS. Rabbit anti-VP6 antibody was used as primary antibody, which was added to the cells for 1 h at 37 °C. Next, the cells were washed three times with PBS. Cell-antibody complexes were then incubated with fluorescein isothiocyanate (FITC)-conjugated goat anti-rabbit IgG (Sigma) for 30 min at 37 °C in the dark. The cells were subsequently washed three times with PBS and observed under a fluorescence microscope. Sp2/0 cells were used as a negative control.

### 2.7. Sequence Alignments and Phylogenetic Analyses

Nt and amino acid sequences of *VP4*, *VP6*, and *VP7* were analyzed using DNASTAR software. The three genes examined in this study and other target gene sequences present in the GenBank database were subjected to genetic and phylogenetic analyses. Multiple nt sequence alignments were performed using Clustal W method. Molecular phylogenetic trees were constructed using the neighbor-joining method with the kimura two-parameter model using MEGA7 software. The bootstrapping probabilities were calculated using 1000 replicates.

## 3. Results

### 3.1. Molecular Characterization

**Figure 2:**
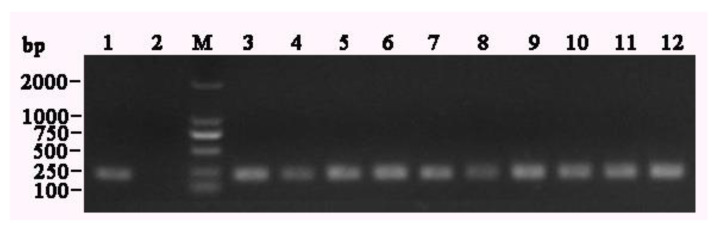
RT-PCR results of the positive samples. (1) NCDV-positive control DNA, (2) negative control, M: Marker DL2000, (3) HLJ8002, (4) HLJ8012, (5) NMG17044, (6) NMG17048, (7) NMG3409, (8) HLJSHJ, (9) JL12031, (10) JL310083, (11) JL40, and (12) JL761565.

Of the 233 fecal samples collected, 10 were identified as BRV-positive using BRV-1/BRV-2 primers by RT-PCR. VP6 gene fragments of 211 bp were amplified (Figure 2). These results were later validated by sequencing. Among them, three samples each were obtained from Heilongjiang Province and Inner Mongolia, whereas four samples were from Jilin Province. RT-PCR was then used to amplify the target genes (VP4, VP6, and VP7).

### 3.2. Virus Isolation 

The MA104 cell line was used to propagate the viruses isolated from all positive samples according to the RT-PCR results. Trypsin-treated samples were used for inoculation of MA104 cells. CPEs were observed as the third virus passage was propagated. Infected cells became round and exhibited clumping at 24 h post-infection (p.i.). The cells became smaller, and the majority of the monolayer was detached at 72 h p.i. Whole-cell lysates were centrifuged, and the supernatant was stored at −80 °C.

### 3.3. Immunoelectron Microscopy

**Figure 3:**
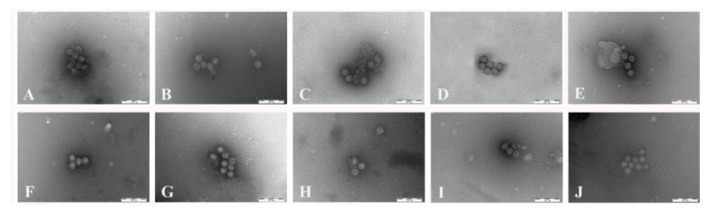
The results of immunoelectron microscopy. (**A**) HLJ8002, (**B**) HLJ8012, (**C**) NMG17044, (**D**) NMG17048, (**E**) NMG3409, (**F**) HLJSHJ, (**G**) JL12031, (**H**) JL310083, (**I**) JL40, (**J**) JL761565.

Cell cultures infected with the ten isolates were treated and incubated with anti-BRV serum. Typical RV particles could be observed using this approach (Figure 3).

### 3.4. Indirect Immunofluorescence Assay

**Figure 4:**
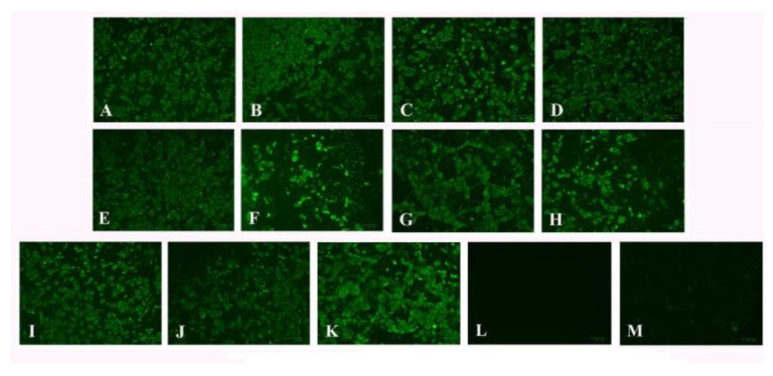
The results of indirect immunofluorescence. (**A**) HLJ8002, (**B**) HLJ8012, (**C**) NMG17044, (**D**) NMG17048, (**E**) NMG3409, (**F**) HLJSHJ, (**G**) JL12031, (**H**) JL310083, (**I**) JL40, (**J**) JL761565, (**K**) NCDV strain, (**L**) MA104 cells infected with NCDV strain and treated with negative serum, and (**M**) non-infected MA104 cells.

To characterize the BRV strains isolated, an indirect immunofluorescence assay was performed. For this, MA104 cells were inoculated with different BRV isolates. After 48 h, the cells were analyzed using the immunofluorescence assay. Obtained results confirmed specific binding of the antibodies to the viral proteins, whereas specific fluorescence signals were absent from control samples (Figure 4).

### 3.5. Sequence Analysis of the Target Genes

**Figure 5:**
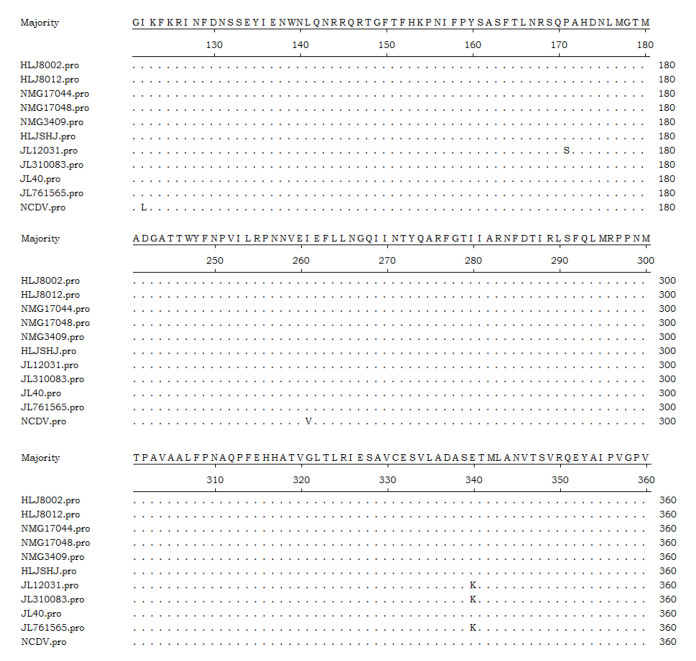
Comparison of deduced aa sequence of *VP6* protein of the isolates with NCDV strain. Dots indicate identical amino acid positions.

The VP6 nt sequences of the isolates and representative type I strains were analyzed for homology. The nt sequence homology of the *VP6* coding region among the ten isolates and NCDV strain (type I2) was above 99.5%, indicating that the ten isolates were all type I2 BRVs. Amino acid sequence comparisons revealed that these isolates were all different from NCDV strain at positions aa122 and aa261 (Figure 5).

**Figure 6:**
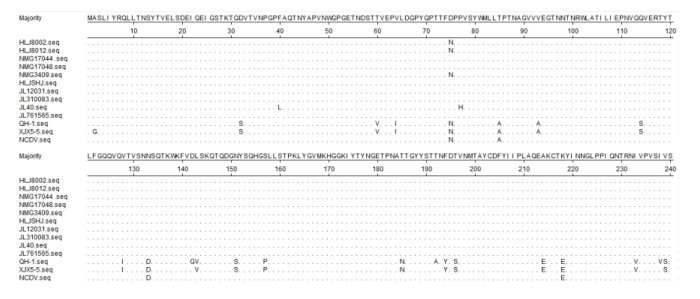

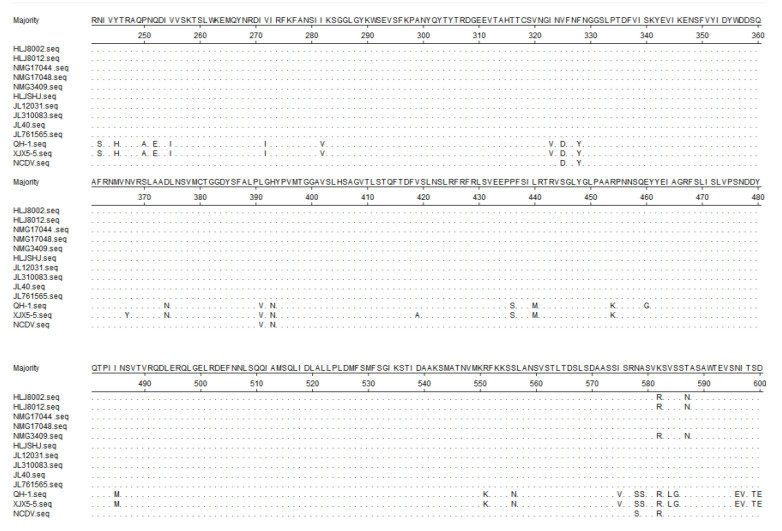
Comparison of deduced aa sequence of *VP4* protein of the isolates with NCDV strain and RVA strains (G6P[1] genotype) detected in China before: QH-1 strain (accession number:MK638873.1); XJX5-5 strain (accession number:MN937514.1). Dots indicate identical amino acid positions.

The VP4 nt sequences of the isolates and other representative P-type strains were analyzed for homology. The *VP4* gene is variable, with 57 different genotypes known. The nt sequence homology between the BRVs isolated in this study, and the NCDV strain was above 99.4%, indicating that the isolated strains were all of the P[1] type. These isolates are all different from the NCDV strain at positions aa85, aa133, aa218, aa325, aa328, aa319, aa393, and aa578. They differed from RVA strains in China reported at many positions, and the results are showed in Figure 6. 

**Figure 7:**
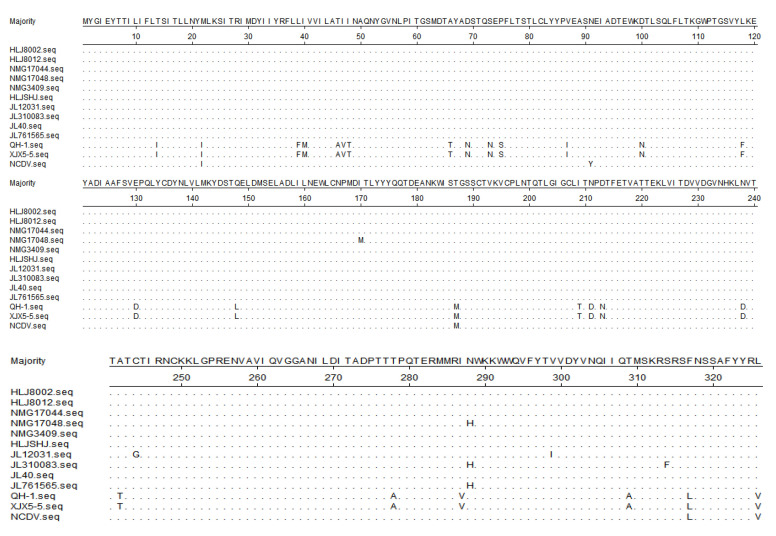
Comparison of deduced aa sequence of *VP7* protein of the isolates with NCDV strain and RVA strains (G6P[1] genotype) detected in China before: QH-1 strain (accession number: MK638875.1); XJX5-5 strain (accession number:MN928498.1). Dots indicate identical amino acid positions.

Further, the *VP7* nt sequences of the isolates and other representative G-type strains were analyzed for homology. The nt sequence homology between the isolates of this study and the NCDV strain in the *VP7* coding region was above 93.4%, and the homology with all other G strains was as high as 77.8%, indicating that all isolates were of the G6 type. The ten isolates were all different from the NCDV strain at positions aa22, aa91, aa187, aa317, and aa326. They differed from RVA strains in China reported at many positions, and the results are showed in Figure 7. 

### 3.6. Phylogenetic Analysis of the Target Genes

**Figure 8:**
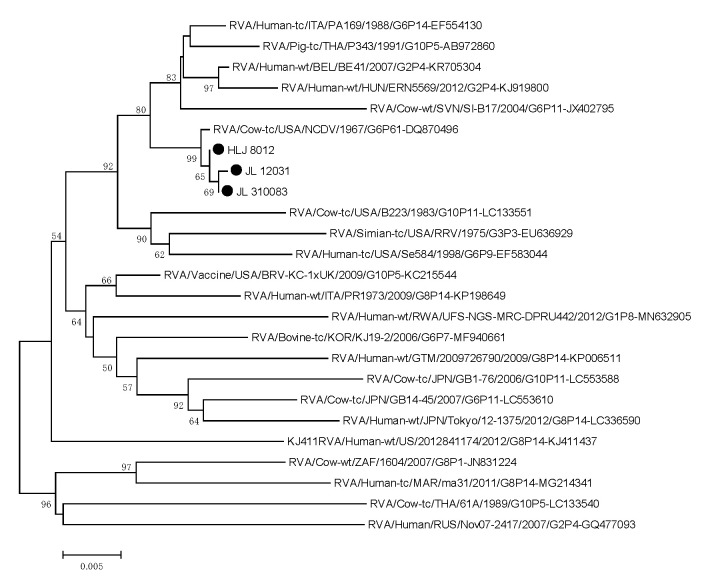
Phylogenetic tree of *VP6* genes.

**Figure 9:**
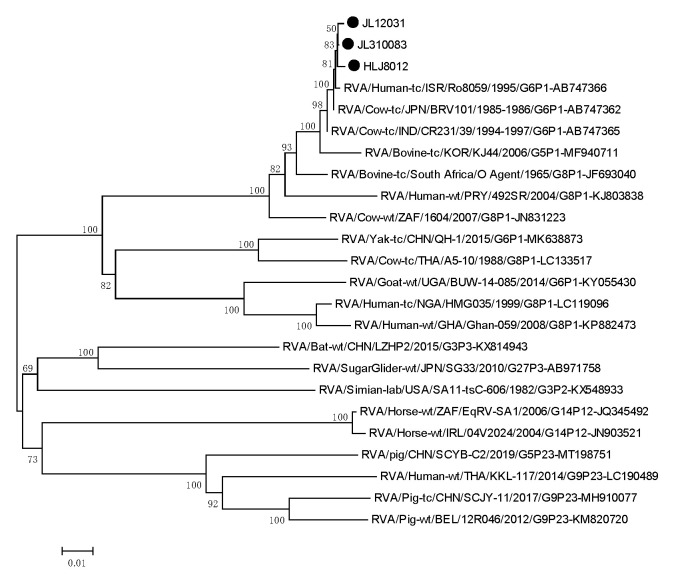
Phylogenetic tree of *VP4* genes.

**Figure 10:**
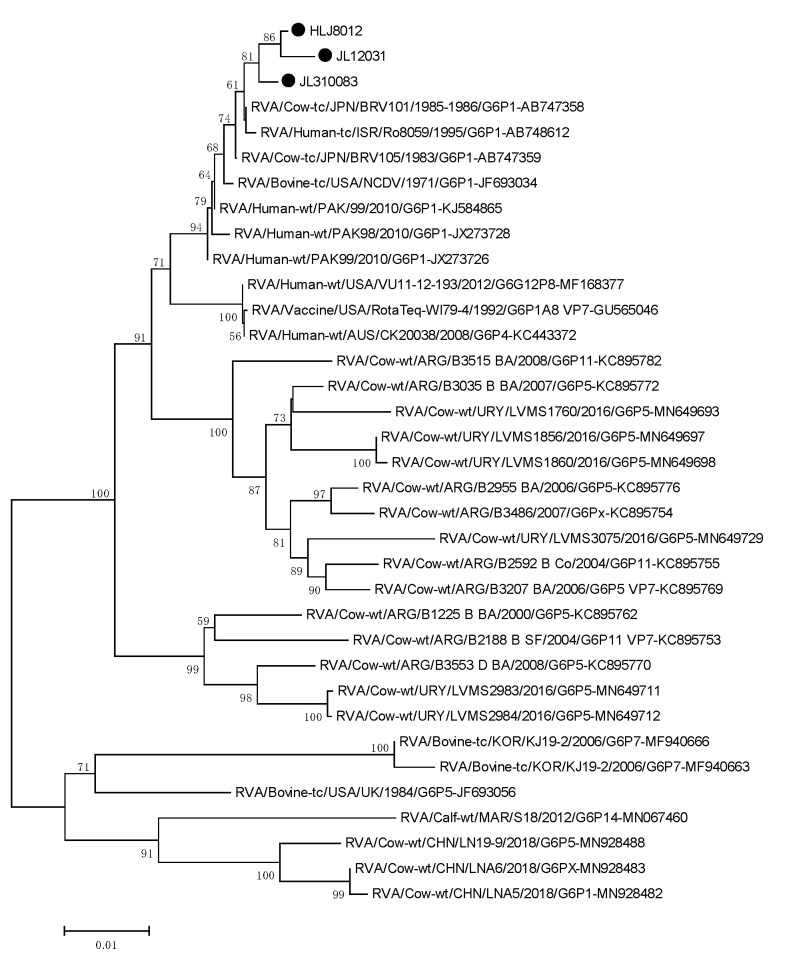
Phylogenetic tree of *VP7* genes.

To investigate the genetic relationship of the isolates and other RVs, we performed phylogenetic analyses using the target genes. For the *VP6* gene, the isolates were clustered with the USA cow RVA NCDV strain (accession number: DQ870496) with 99.63–99.85% sequence identity. The VP4 genes of the isolates showed the highest sequence identity (99.47–99.60%) with the Israelian human RVA G6P[1] strain (accession number: AB747366). The isolates were shown to be closely related to the Ro8095 strain, which is found in a common branch with several bovine and bovine-like strains from Asia. The VP7 genes of the isolates were closely related to Japanese cow RVA BRV101 of the G6P[1] strain (accession number: AB747358), with which they shared the highest nt sequence identity (99.45–99.78%) (Figure 8, Figure 9 and Figure 10).

Phylogenetic tree of the *VP6* genes, reconstructed using the sequence of BRA HLJ8021, JL12021, JL210083 strain, ia represented by a filled circle. The numbers adjacent to the nodes represent the percentage of bootstrap support (1000 replicates) for the clusters. Bootstrap values less than 50% are not shown.

Phylogenetic tree of the *VP4* genes, reconstructed using the sequence of genotype P[1] of the BRA HLJ8021, JL12021, JL210083 strain, is represented by a filled circle. The numbers adjacent to the nodes represent the percentage of bootstrap support (1000 replicates) for the clusters. Bootstrap values less than 50% are not shown.

Phylogenetic tree of the *VP7* genes, reconstructed using the sequence of genotype G6 of the BRA HLJ8021, JL12021, JL210083 strain, is represented by a filled circle. The numbers adjacent to the nodes represent the percentage of bootstrap support (1000 replicates) for the clusters. Bootstrap values less than 50% are not shown.

## 4. Discussion

RVs are important pathogens causing diarrhea in calves. The prevalence of BRV varies worldwide. RVA was previously identified in 25.4% (*n* = 17) of 67 diarrheic fecal samples evaluated in Brazil [19]. In India, three out of 45 samples (6.66%) from necropsied calves were positive for BRV [28]. In 2020, fecal samples of calves collected from 39 cattle farms in northwest Argentina between 2014 and 2016 were analyzed. The results revealed that BRV infections were detected in 20 of the 39 cattle farms, with a positive rate of 8.4% (67/795) [29]. Therefore, BRV is still one of the main etiological agents of neonatal diarrhea in calves worldwide. Morbidity and mortality rates associated with BRV infections are high, which in turn cause important direct and indirect economic losses to beef and dairy production. 

The current study highlights that BRV continues to circulate within calves with symptomatic diarrhea in northeast China, according to data collected between 2017 and 2020. In this study, 233 bovine diarrheic samples were collected from modern cattle farms with good sanitation and regular vaccination, which were located in Heilongjiang and Jilin Provinces and the Inner Mongolia Autonomous Region. Of these, 78, 81, and 74 samples were obtained from the Inner Mongolia Autonomous Region, Heilongjiang Province, and Jilin Province, respectively. Diarrhea in calves occurred mainly in fall and winter on the different cattle farms, with an age range of 3–8 weeks. After screening of fecal samples from diarrheic cases by RT-PCR, positive samples were used to infect MA104 cells. The results showed that ten samples were BRV-positive, resulting in a positive rate of 4.29%. These included three samples from the Inner Mongolia Autonomous Region (from two farms), three from Heilongjiang Province (from one farm), and four from Jilin Province (from two farms). Other studies on the prevalence of BRV have been conducted previously in China. In Yangxin County, China, 69 fecal samples were collected from diarrheic newborn calves and analyzed for BRV. RT-PCR results showed that the percentage of BRV-positive samples was 36.23% [30]. A previous study conducted between 2018 and 2019 reported an overall prevalence of RV in newborn calves of 20.0% (15/75) in Hebei Province, China [31]. Compared to previous studies, the BRV-positive rate obtained in this study was relatively low (6.66%). The reduction in BRV prevalence may be attributed to the popularity of vaccination in the regions analyzed. Although there has not been a BRV diarrhea epidemic recently, there are still a certain degree of positive cases. It is speculated that some of the epidemic BRV strains have diverged from traditional strains used for vaccines.

BRV is a common infectious agent leading to NCD [32,33], which often occurs in combination with other pathogens. Notably, a combination of viral and protozoan pathogens occurred frequently (37.5%) in the samples analyzed, followed by the combination of viral and bacterial (25%) as well as viral, bacterial, and protozoan (25%) infectious agents, which was contrasted by fewer cases being caused by viral agents alone (12.5%) [34]. Brunauer and colleagues carried out 41 studies in 21 countries to determine the presence or absence of mixed infections in calves globally. The results showed that the highest pooled prevalence was identified for BRV-Crypto (6.69%), followed by BRV-BCoV (2.84%), and BRV-ETEC (1.64%) [35]. A synergistic effect was also observed between BRV and BCoV. In this study, among the 233 diarrheal samples examined, the BRV-positive rate was 4.29%, including 10% of cases with mixed BRV and bovine parvovirus (BPV) infections. BPV infections mainly cause respiratory and gastrointestinal diseases in newborn calves. Synergistic effects are known to occur in mixed BRV-BPV infections, which may cause more severe diarrheal symptoms in calves. 

VP6 is the major structural component of the RV capsid and plays an important role in defining the virion structure [3]. It is considered to be the most strongly conserved RV protein, showing little variability among the same group of RVs. At present, RVA is the most common group. Within this group, viruses are divided into different serotypes, of which I2, I3, and I5 are the major serotypes. Type I2 BRV is prevalent worldwide. The ten strains isolated in this study were all type I2 strains. The VP6 sequences were closely related to the USA cow RVA NCDV strain (RVA/cow-tc/USA/NVDV/1967/G6P[1]). *VP6* genes were analyzed based on amino acid sequence comparisons. The results showed that some amino acid residues had changed and that all strains isolated differed from the NCDV strain at positions aa122 and aa261. Furthermore, aa261, an ectopic site, was previously found to be mutated. Max Ciarlet et al. found that the BRV strain WC3 isolated by them was also different from the NCDV strain at position aa261 [36]. In addition, the other site identified in this study (aa122) is located in the main polymerization region of *VP6* (122–147). As the *VP6* gene is the most commonly used target for BRV detection, the impact of the resulting amino acid change on BRV detection methods deserves attention.

*VP4* is the spike protein of RV, which is associated with hemagglutination, neutralization, and infectivity. It can be cleaved into *VP8* (aa1–aa240) and VP5 (aa248–aa775) by trypsin. Studies using neutralizing monoclonal antibodies have identified eight neutralization epitopes on VP4. Five of those are located in the VP8 subunit of VP4 [37,38,39]. The VP4 sequences obtained in this study were closely related to those of the Japanese BRV strain (RVA/cow-tc/JPN/BRV101/1985–1986/G6P[1]). VP4 serotypes are categorized based on the primary structure of the amino acid sequence of the VP8 fragment. The *VP4* genes of the isolates were analyzed by amino acid sequence comparisons. The results showed that the HLJ8002, HLJ8012, NMG17044, NMG3409, and HLJ SHJ strains were mutated at positions aa85, aa133, and aa218. NMG17048 and JL12031 strains were mutated at positions aa75, aa85, aa133, and aa218. JL310083 and JL761565 strains displayed mutations at positions aa75, aa77, aa85, aa133, and aa218. These mutated residues were all located within the VP8 fragment, which may have contributed to the altered immunogenicity. 

The *VP7* protein plays a major role in virus stability and particle formation of RV [3]. It elicits the production of neutralizing antibodies and defines the major antigenic specificities due to which neutralizing immune responses are mounted during RV infections [40]. The main neutralizing epitopes of *VP7* are found at positions aa87-aa101, aa145-aa150, and aa208-aa221. Jalilian et al. investigated potential epitopes present in *VP7* and concluded that amino acid residues aa87–93, aa87–100aa, and aa79–93 likely serve as B- and T-cell epitopes [41]. The *VP7* sequences of the isolates were closely related to a human strain from Israel (RVA/Human-tc/ISR/ro8059/1995/G6P[1]). All isolates were mutated at position aa91, which resides in the B- and T-cell epitopes predicted by Jalilian et al. These results indicate that the antigenic sites of the isolates had already changed compared to the traditional strain. Although the regular vaccinations of BRV strain (G6P[1] genotype) have been used in these farms, the diarrheal symptoms still persisted in the herds. It suggests these mutations may affect the immunogenicity of the BRV isolates. It is unclear whether antigenic mismatches between vaccine antigens and field viruses also affect vaccine efficacy. Epidemiological data on antigenic heterogeneity of the BRVs isolated will be useful for understanding the patterns of antigenic site replacement and may help improve vaccination strategies. 

With ongoing cattle industry development worldwide, the serotypes of epidemic BRV strains have begun to diversify. Two G (G6 and G10) and two P (P[5] and P[11]) types were predominant among the RVA strains isolated [1,17,23,42,43,44]. In China, G6P[1] was found to be the most frequent variant in calves [27,45]. In this study, nt sequence analysis of the *VP4* and *VP7* genes present in the isolates revealed that they were all of the G6P[1] genotype, confirming that the G6P[1] variant predominates in China.

## 5. Conclusions

In conclusion, ten BRV strains were isolated from calves in northeast China. The epidemiological characteristics and genetic diversity of these isolates were investigated, which indicated that the antigenicity and infectivity of the isolates was altered compared to the traditional strain. These findings could prove useful for understanding the epidemiology of BRV in China and for designing an effective vaccine to control BRV infections. However, the significance of this study is limited by the fact that the samples were only collected from the regions of Heilongjiang Province, Jilin Province, and the Inner Mongolia Autonomous Region of China. Therefore, further studies in other areas of China are required. Moreover, for a comprehensive analysis of the genetic diversity of RVA, G, and P genotypes of bovine RVA strains circulating during outbreaks of NCD in vaccinated cattle herds should be screened on a regular basis.

## Figures and Tables

**Table 1:** Sample collection information.

Sample Area	Number of Farms	Number of Samples	Health Management	Age of the Cattles
Heilongjiang	3	81	Diarrhea	3–8 weeks
Neimenggu	5	78	Diarrhea	3–8 weeks
Jilin	4	74	Diarrhea	3–8 weeks

**Table 2:** Primers sequence used for RT-PCR in the present survey.

Name	Primers (5′–3′)	Product Size (bp)
BRV-1	CGATAATGTATGTATGGACG	211
BRV-2	TGCTGAATAAGGGAAAATG	
BRV-VP4-F	GGCTTTAAAATGGCTTCACTCAT	2362
BRV-VP4-R	GGTCACATCCTCTGTCAGTTGCT	
BRV-VP6-F	GGCTTTTAAACGAAGTCTTCA	1356
BRV-VP6-R	GGTCACATCCTCTCACTACG	
BRV-VP7-F	GGCTTTAAAAGCGAGAATTTCCGTT	1062
BRV-VP7-R	GGTCACATCATACAACTCTAACT	

## Data Availability

De-identified data are available after personal email request to the authors Xi Cheng (18008349515@163.com) and Xinyuan Qiao (qiaoxinyuan@126.com).

## References

[1] Elkady G., Zhu J., Peng Q., Chen M., Guo A. (2021). Isolation and whole protein characterization of species A and B bovine rotaviruses from Chinese calves. Infect. Genet. Evol..

[2] Matthijnssens J., Otto P.H., Ciarlet M., Desselberger U., Ranst M.V., Johne R. (2012). VP6-sequence-based cutoff values as a criterion for rotavirus species demarcation. Arch. Virol..

[3] Estes M.K., Greenberg H.B. (2013). Rotaviruses. Fields Virology.

[4] Matthijnssens J.M., Ciarlet S.M., Mcdonald H., Attoui K., Bányai J.R., Brister J., Buesa M.D., Esona M.K., Estes J.R. (2011). Gentsch Uniformity of Rotavirus Strain Nomenclature Proposed by the Rotavirus Classification Working Group (RCWG). Arch. Virol..

[5] Snodgrass D.R., Terzolo H.R., Sherwood D., Campbell I., Menzies J.D., Synge B.A. (1986). Aetiology of diarrhoea in young calves. Vet. Rec..

[6] Windeyer M.C., Leslie K.E., Godden S.M., Hodgins D.C., Lissemore K.D., Leblanc S.J. (2014). Factors associated with morbidity, mortality, and growth of dairy heifer calves up to 3 months of age. Prev. Vet. Med..

[7] Karayel I., Marton S., Coskun N., Bányai K., Alkan F. (2017). Putative vaccine breakthrough event associated with heterotypic rotavirus infection in newborn calves, Turkey, 2015. Vet. Microbiol..

[8] Alkan F., Ozkul A., Oguzoglu T.C., Timurkan M.O., Caliskan E., Martella V., Burgu I. (2010). Distribution of G (VP7) and P (VP4) genotypes of group A bovine rotaviruses from Turkish calves with diarrhea, 1997–2008. Vet. Microbiol..

[9] Swiatek D.L., Palombo E.A., Lee A., Coventry M.J., Britz M.L., Kirkwood C.D. (2010). Detection and analysis of bovine rotavirus strains circulating in Australian calves during 2004 and 2005. Vet. Microbiol..

[10] Badaracco A., Garaicoechea L., Matthijnssens J., Uriarte E.L., Odeón A., Bilbao G., Fernandez F., Parra G.I., Parre O.V. (2013). Phylogenetic analyses of typical bovine rotavirus genotypes G6, G10, P[5] and P[11] circulating in Argentinean beef and dairy herds. Infect. Genet. Evol..

[11] Medeiros T.S., Lorenzetti E., Alfieri A.F., Alfieri A.A. (2015). Phylogenetic analysis of a G6P[5] bovine rotavirus strain isolated in a neonatal diarrhea outbreak in a beef cattle herd vaccinated with G6P[1] and G10P[11] genotypes. Arch. Virol..

[12] Cortese V.S. (2009). Neonatal immunology. Vet. Clin. N. Am. Food Anim. Pract..

[13] Bartels C., Holzhauer M., Jorritsma R., Swart W., Lam T. (2010). Prevalence, prediction and risk factors of enteropathogens in normal and non-normal faeces of young Dutch dairy calves. Prev. Vet. Med..

[14] Meganck V., Hoflack G., Piepers S., Opsomer G. (2015). Evaluation of a protocol to reduce the incidence of neonatal calf diarrhoea on dairy herds. Prev. Vet. Med..

[15] Matthijnssens J., Bilcke J., Ciarlet M., Martella V., Bányai K., Rahman M., Zeller M., Beutels P., Damme P.V., Ranst M.V. (2009). Rotavirus disease and vaccination: Impact on genotype diversity. Future Microbiol..

[16] Martella V., Bányai K., Matthijnssens J., Buonavoglia C., Ciarlet M. (2010). Zoonotic aspects of rotaviruses. Vet. Microbiol..

[17] Badaracco A., Garaicoechea L., Rodríguez D., Uriarte E.L., Odeón A., Bilbao G., Galarza R., Abdala A., Fernandez F., Parre O.V. (2012). Bovine rotavirus strains circulating in beef and dairy herds in Argentina from 2004 to 2010. Vet. Microbiol..

[18] Usonis V., Ivaskeviciene I., Desselberger U., Rodrigo C. (2012). The unpredictable diversity of co-circulating rotavirus types in Europe and the possible impact of universal mass vaccination programmes on rotavirus genotype incidence. Vaccine.

[19] Fritzen J., Lorenzetti E., Oliveira M.V., Bon V.R., Ayres H., Alfieri A.F., Alfieri A.A. (2019). Cross-sectional study of the G and P genotypes of rotavirus A field strains circulating in regularly vaccinated dairy cattle herds. Trop. Anim. Health Prod..

[20] Matthijnssens J., Ciarlet M., Heiman E., Arijs I., Delbeke T., McDonald S.M., Palombo E.A., Iturriza-Gomara M., Maes P., Patton J.T. (2008). Full Genome-Based Classification of Rotaviruses Reveals a Common Origin between Human Wa-Like and Porcine Rotavirus Strains and Human DS-1-Like and Bovine Rotavirus Strains. J. Virol..

[21] Rahman M., Banik S., Faruque A., Taniguchi K., Sack D.A., Van Ranst M., Azim T. (2005). Detection and characterization of human group C rotaviruses in Bangladesh. J. Clin. Microbiol..

[22] Hussein H.A., Parwani A.V., Rosen B.I., Lucchelli A., Saif L.J. (1993). Detection of rotavirus serotypes G1, G2, G3, and G11 in feces of diarrheic calves by using polymerase chain reaction-derived cDNA probes. J. Clin. Microbiol..

[23] Garaicoechea L., Bok K., Jones L.R., Combessies G., Odeón A., Fernandez F., Parre O.V. (2006). Molecular characterization of bovine rotavirus circulating in beef and dairy herds in Argentina during a 10-year period (1994–2003). Vet. Microbiol..

[24] Park S.H., Saif L.J., Jeong C., Lim G.K., Park S.I., Kim H.H., Park S.J., Kim Y.J., Jeong J.H., Kang M.I. (2006). Molecular characterization of novel G5 bovine rotavirus strains. J. Clin. Microbiol..

[25] Collins P.J., Cullinane A., Martella V., O’Shea H. (2008). Molecular characterization of equine rotavirus in Ireland. J. Clin. Microbiol..

[26] Malik Y.S., Sharma K., Vaid N., Chakravarti S., Chandrashekar K.M., Basera S.S., Singh R., Prasad G., Gulati B.R., Bhilegaonkar K.N. (2012). Frequency of group A rotavirus with mixed G and P genotypes in bovines: Predominance of G3 genotype and its emergence in combination with G8/G10 types. J. Vet. Sci..

[27] Yan N., Yuanwei W., Bin Z., Hua Y., Cheng T. (2020). High prevalence and genomic characteristics of G6P[1] Bovine Rotavirus A in yak in China. J. Gen. Virol..

[28] Singh S., Singh R., Singh K.P., Singh V., Malik Y., Kamdi B., Singh R., Kashyap G. (2020). Immunohistochemical and molecular detection of natural cases of bovine rotavirus and coronavirus infection causing enteritis in dairy calves. Elsevier Public Health Emerg. Collect..

[29] Bertoni E., Aduriz M., Bok M., Vega C., Saif L., Aguirre D., Cimino R.O., Miño S., Parreño V. (2020). First report of group A rotavirus and bovine coronavirus associated with neonatal calf diarrhea in the northwest of Argentina. Trop. Anim. Health Prod..

[30] Wei X., Wang W., Dong Z., Cheng F., Zhang J. (2021). Detection of Infectious Agents Causing Neonatal Calf Diarrhea on Two Large Dairy Farms in Yangxin County, Shandong Province, China. Front. Vet. Sci..

[31] Zhang Z., Su D., Meng X., Liang R., Feng Y. (2021). Cryptosporidiosis outbreak caused by Cryptosporidium parvum subtype IIdA20G1 in neonatal calves. Transbound Emerg. Dis..

[32] Gillhuber J., Rügamer D., Pfister K., Scheuerle M. (2014). Giardiosis and other enteropathogenic infections: A study on diarrhoeic calves in Southern Germany. BMC Res. Notes.

[33] Uhde F.L., Kaufmann T., Sager H., Albini S., Zanoni R., Schelling E., Meylan M. (2008). Prevalence of four enteropathogens in the faeces of young diarrhoeic dairy calves in Switzerland. Vet. Rec. J. Br. Vet. Assoc..

[34] Dall Agnol A.M., Lorenzetti E., Leme R.A., Ladeia W.A., Mainardi R.M., Bernardi A., Headley S.A., Freire R.L., Pereira U.P., Alfieri A.F. (2021). Severe outbreak of bovine neonatal diarrhea in a dairy calf rearing unit with multifactorial etiology. Brazilian journal of microbiology. Braz. Soc. Microbiol..

[35] Brunauer M., Roch F.F., Conrady B. (2021). Prevalence of Worldwide Neonatal Calf Diarrhoea Caused by Bovine Rotavirus in Combination with Bovine Coronavirus, Escherichia coli K99 and Cryptosporidium spp.: A Meta-Analysis. Animals.

[36] Ciarlet M., Hyser J.M., Estes M.K. (2002). Sequence analysis of the VP4, VP6, VP7, and NSP4 gene products of the bovine rotavirus WC3. Virus Genes.

[37] Favacho A.R.M., Kurtenbach E., Sardi S.I., Gouvea V.S. (2006). Cloning, expression, and purification of recombinant bovine rotavirus hemagglutinin, VP8*, in Escherichia coli. Protein Expr. Purif..

[38] Fernandez F.M., Conner M.E., Hodgins D.C., Parwani A.V., Nielsen P.R., Crawford S.E., Estes M.K., Saif L.J. (1998). Passive immunity to bovine rotavirus in newborn calves fed colostrum supplements from cows immunized with recombinant SA11 rotavirus core-like particle (CLP) or virus-like particle (VLP) vaccines. Vaccine.

[39] Greenberg H.B., Valdesuso J.O.S.E., van Wyke K.A.T.H.L.E.E.N., Midthun K.A.R.E.N., Walsh M., McAuliffe V., Wyatt R.G., Kalica A.R., Flores J., Hoshino Y. (1983). Production and preliminary characterization of monoclonal antibodies directed at two surface proteins of rhesus rotavirus. J. Virol..

[40] Aminu M., Page N.A., Ahmad A.A., Umoh J.U., Dewar J., Steele A.D. (2010). Diversity of Rotavirus VP7 and VP4 Genotypes in Northwestern Nigeria. J. Infect. Dis..

[41] Jalilian S., Teimoori A., Makvandi M. (2019). In Silico Characterization of Epitopes from Human Rotavirus VP7 Genotype G9 Design for Vaccine Development. Iran. J. Allergy Asthma Immunol..

[42] Midgley S.E., Bányai K., Buesa J., Halaihel N., Bttiger B. (2011). Diversity and zoonotic potential of rotaviruses in swine and cattle across Europe. Vet. Microbiol..

[43] Alfieri A.F., Alfieri A.A., Barreiros M., Leite J., Richtzenhain L.J. (2004). G and P genotypes of group A rotavirus strains circulating in calves in Brazil, 1996–1999. Vet. Microbiol..

[44] Reidy N., Lennon G., Fanning S., Power E., O’Shea H. (2006). Molecular characterisation and analysis of bovine rotavirus strains circulating in Ireland 2002–2004. Vet. Microbiol..

[45] Xiaoying L., Nan Y., Hua Y., Yuanwei W., Bin Z., Cheng T. (2021). Detection and molecular characteristics of bovine rotavirus A in dairy calves in China. J. Vet. Sci..

